# Deleterious effects of a combination therapy using fluoroquinolones and tetracyclines for the treatment of Japanese spotted fever: a retrospective cohort study based on a Japanese hospital database

**DOI:** 10.1093/jac/dkae192

**Published:** 2024-06-12

**Authors:** Ikkoh Yasuda, Michiko Toizumi, Eiichiro Sando

**Affiliations:** Department of General Internal Medicine and Clinical Infectious Diseases, Fukushima Medical University, Fukushima, Japan; Department of General Internal Medicine and Infectious Diseases, Kita-Fukushima Medical Center, Fukushima, Japan; Department of Paediatric Infectious Diseases, Institute of Tropical Medicine, Nagasaki University, Nagasaki, Japan; Department of General Internal Medicine and Clinical Infectious Diseases, Fukushima Medical University, Fukushima, Japan; Department of General Internal Medicine and Infectious Diseases, Kita-Fukushima Medical Center, Fukushima, Japan

## Abstract

**Objectives:**

Tetracyclines are the standard treatment for rickettsiosis, including Japanese spotted fever (JSF), a tick-borne rickettsiosis caused by *Rickettsia japonica*. While some specialists in Japan advocate combining fluoroquinolones with tetracyclines for treating JSF, the negative aspects of combination therapy have not been thoroughly evaluated. Whether fluoroquinolones should be combined with tetracyclines for JSF treatment is controversial. The study aimed to evaluate the disadvantages of fluoroquinolones combined with tetracyclines for JSF treatment.

**Methods:**

This retrospective cohort study was conducted using a Japanese database comprising claims data from April 2008 to December 2020. The combination therapy group (tetracyclines and fluoroquinolones) was compared with the monotherapy group (tetracycline only) regarding mortality and the incidence of complications.

**Results:**

A total of 797 patients were enrolled: 525 received combination therapy, and 272 received monotherapy. The adjusted odds ratio (OR) for mortality was 2.30 [95% confidence interval (CI): 0.28–18.77] in the combination therapy group with respect to the monotherapy group. According to the subgroup analysis, patients undergoing combination therapy with ciprofloxacin experienced higher mortality rates compared with those receiving monotherapy (adjusted OR = 25.98, 95% CI = 1.71–393.75). Additionally, 27.7% of the combination therapy group received NSAIDs concurrently with fluoroquinolones. The combination therapy with NSAIDs group was significantly more likely to experience convulsions than the monotherapy without NSAIDs group (adjusted OR: 5.44, 95% CI: 1.13–26.30).

**Conclusions:**

This study found no evidence that combination therapy improves mortality outcomes and instead uncovered its deleterious effects. These findings facilitate a fair assessment of combination therapy that includes consideration of its disadvantages.

## Introduction

Japanese spotted fever (JSF), a tick-borne disease caused by *Rickettsia japonica*, is endemic in Japan.^1^ According to reports, the number of JSF cases in Japan increased from 135 in 2008 to 318 in 2019, and the case fatality rate increased from 0.7% to 4.1%.^2,3^ Tetracyclines (TCs) are the standard treatment for JSF, and some experts in Japan recommend the use of a fluoroquinolone (FQ) in addition to a TC with the expectation of a synergistic effect.^4,5^ However, there are no reported improvements in mortality with the addition of FQs to TCs,^6^ and the validity of this combination therapy has not been sufficiently verified.

On the other hand, the negative aspects of combination therapy have not been thoroughly evaluated, although it is essential to assess both the positive and negative aspects of using FQs in combination with TCs to evaluate the validity of this type of therapy. FQs are also known to cause adverse effects, including convulsions, upper gastrointestinal ulcers, diarrhoea, arrhythmia and hypoglycaemia.^7^ Concurrent use of nonsteroidal anti-inflammatory drugs (NSAIDs) and FQs can further increase the likelihood of convulsions.^8^ Diseases such as Rocky Mountain spotted fever and Mediterranean spotted fever, which are classified as spotted fever group rickettsioses, similar to JSF, are not recommended for monotherapy with FQs due to the lack of human efficacy data and their association with delayed defervescence and increased disease severity.^9–11^ Careful consideration is needed especially for the use of FQs in the treatment of JSF.

The aim of this study was to assess the disadvantages of using FQs in addition to TCs for JSF treatment. To achieve this objective, we assessed the side effects associated with combination therapy during JSF treatment using information from a Japanese database.

## Materials and methods

A retrospective cohort study was conducted using a Japanese database provided by Medical Data Vision Co., Ltd. (MDV database) from April 2008 to December 2020.^12^ As of January 2019, the MDV database included claims data from approximately 26 million patients who were treated in 380 acute care hospitals designated as diagnosis procedure combination (DPC) hospitals. DPC is a system for categorizing medical care that was developed in Japan,^13^ and anonymized clinical data from DPC hospitals are made available for research purposes.

### Definitions of target cases, treatment and severity

The inclusion criterion was at least one confirmed diagnosis of JSF, identified with disease code 8838411 in the MDV database, during the study period; patients with a suspected diagnosis of JSF were excluded. Patients who received only a TC within one month of receiving their JSF diagnosis were categorized into the monotherapy group. Patients who received both an FQ and a TC within one month of receiving their JSF diagnosis and who used both drugs concurrently for more than one day were categorized into the combination therapy group. Patients who switched and used an FQ and a TC without overlapping usage were excluded. Patients who used NSAIDs concurrently with TCs in the monotherapy group or with FQs in the combination therapy group for more than one day were considered to have concomitant NSAID use. Severe JSF was clinically defined as having at least one of the following diagnoses involving organ dysfunctions or conditions requiring intensive care in the same month as the diagnosis: renal failure, respiratory failure, meningitis, altered mental status and/or vasopressor use.

The primary outcomes were the incidence of complications, including mortality, convulsions, upper gastrointestinal ulcers, diarrhoea, arrhythmia and hypoglycaemia that occurred within one month after the onset of JSF.

### Statistical analysis

The demographic characteristics and proportions of hospitalized or severe JSF patients in the therapy groups were compared using Fisher’s exact test for categorical variables or the Mann–Whitney U test for continuous variables. Using logistic regression, we calculated the adjusted odds ratios (ORs) and corresponding 95% confidence intervals (CIs) for the effects of combination therapy with respect to those of monotherapy on the outcomes of mortality, convulsions, upper gastrointestinal ulcers, diarrhoea, arrhythmia and hypoglycaemia after controlling for potential confounding factors such as sex, age group and severity. For mortality, if follow-up data were available after the first month following a JSF diagnosis, survival was assumed. Missing values related to mortality were addressed using multiple imputation. We also conducted subgroup analyses in which logistic regression was also used to analyse the effects of the type of FQ, including levofloxacin (LVFX) and ciprofloxacin (CPFX), on each outcome and the effects of NSAID use in monotherapy and combination therapy on the development of convulsions. To determine whether the effect of combination therapy on convulsions was modified by the use of NSAIDs, we included an interaction term between treatment type (monotherapy versus combination therapy) and NSAID use in the multivariable model. A *P* value less than 0.05 was considered to indicate statistical significance. All analyses were performed using Stata version 18.0 (Stata Corp., College Station, TX, USA).

### Ethics

The study was approved by the Institutional Review Board of Fukushima Medical University (2022-166). Due to the anonymous nature of the data, no identifiable patient information was acquired, and the requirement for written informed consent was waived by the ethics committee.

## Results

A total of 797 patients were enrolled. The basic characteristics of the participants at the time of diagnosis are summarized in Table 1. A total of 525 (65.9%) patients were categorized into the combination therapy group, and 272 (34.1%) were categorized into the monotherapy group. The median age of all participants was 71 years [interquartile range (IQR): 61–79 years], and 549 patients (68.9%) were 65 years old or older. A total of 679 (85.2%) patients were inpatients, and 114 (14.3%) had severe disease. The median age of the combination therapy group was greater than that of the monotherapy group [median age of 73 years (IQR: 64–80) versus 67 years (IQR: 53–77); *P* < 0.001], and the combination therapy patients were more likely to be 65 years or older than the monotherapy patients [389 (74.1%) versus 160 (58.8%); *P* < 0.001]. Compared with those in the monotherapy group, the patients in the combination therapy group were more likely to be hospitalized [487 (92.8%) versus 192 (70.6%); *P* < 0.001] and to have severe disease [97 (18.5%) versus 17 (6.2%); *P* < 0.001].

**Table 1.** Basic characteristics of the study participants at the time of JSF diagnosis

	Total (*N* = 797)	Monotherapy (*N* = 272)	Combination therapy (*N* = 525)	*P* value
Sex (female), *n* (%)	415 (52.1%)	134 (49.3%)	281 (53.5%)	0.26
Age				
Median age, years (IQR)	71 (61–79)	67 (53–77)	73 (64–80)	<0.001
Age ≥65 years, *n* (%)	549 (68.9%)	160 (58.8%)	389 (74.1%)	<0.001
Inpatient, *n* (%)	679 (85.2%)	192 (70.6%)	487 (92.8%)	<0.001
Severe cases, *n* (%)	114 (14.3%)	17 (6.2%)	97 (18.5%)	<0.001

IQR, interquartile range; Monotherapy, patients who received only a TC within one month of their JSF diagnosis; Combination therapy, patients who received both an FQ and a TC within one month of their JSF diagnosis and who used both drugs concurrently for more than one day. Patient characteristics were compared between the therapy groups using Fisher’s exact test for categorical variables or the Mann–Whitney U test for continuous variables.

To evaluate the positive and negative aspects of using FQs in combination with TCs, the incidence of each outcome, including mortality and complications, was compared between the monotherapy group and combination therapy group. Table 2 shows the number of patients, ORs and 95% CIs for the combination therapy group corresponding to each outcome. Of 797 patients, 55 (6.9%) had missing values related to mortality, which were addressed using multiple imputation. The likelihood of mortality tended to be greater in the combination therapy group than in the monotherapy group, although the difference was not statistically significant [adjusted odds ratio (OR) 2.30, 95% CI = 0.28–18.77]. No associations were detected between the type of therapy (combination therapy versus monotherapy) and any complications, including convulsions, upper gastrointestinal ulcers, diarrhoea, arrhythmia or hypoglycaemia.

**Table 2.** ORs and 95% CIs of tetracycline and FQ combination therapy for each clinical outcome with respect to tetracycline monotherapy

	*n* (%)	Unadjusted OR^a^	Adjusted OR^b^
Mortality			
Monotherapy (*N* = 272)	1 (0.4%)	1 (reference)	1 (reference)
Combination therapy (*N* = 525)	10 (2.0%)	4.94 (0.64–37.87)	2.30 (0.28–18.77)
Convulsions			
Monotherapy	4 (1.5%)	1 (reference)	1 (reference)
Combination therapy	19 (3.6%)	2.52 (0.85–7.47)	2.14 (0.70–6.48)
Upper gastrointestinal ulcers			
Monotherapy	22 (8.1%)	1 (reference)	1 (reference)
Combination therapy	63 (12.0%)	1.55 (0.93–2.58)	1.18 (0.69–2.00)
Diarrhoea			
Monotherapy	5 (1.8%)	1 (reference)	1 (reference)
Combination therapy	7 (1.3%)	0.72 (0.23–2.30)	0.42 (0.12–1.48)
Arrhythmia			
Monotherapy	5 (1.8%)	1 (reference)	1 (reference)
Combination therapy	19 (3.6%)	2.01 (0.74–5.43)	1.62 (0.58–4.52)
Hypoglycaemia			
Monotherapy	2 (0.7%)	1 (reference)	1 (reference)
Combination therapy	2 (0.4%)	0.52 (0.07–3.69)	0.38 (0.05–2.82)

A logistic regression model was used to calculate the adjusted OR with the 95% CI for mortality or complications by controlling for the above potential confounding factors. OR, odds ratio; Monotherapy, patients who received only TC within a month of their JSF diagnosis; Combination therapy, patients who received both an FQ and a TC within one month of their JSF diagnosis and who used both drugs concurrently for more than one day.

^a^Unadjusted OR.

^b^ORs adjusted for sex, age group and severity.

We then conducted subgroup analysis to explore the differences in outcome incidence based on the type of FQ used for combination therapy. Among the 525 patients in the combination therapy group, the FQs used were as follows: LVFX in 457 patients (87.0%), CPFX in 30 (5.7%), pazufloxacin in 10 (1.9%), tosufloxacin in 6 (1.1%), sitafloxacin in 2 (0.4%) and the coadministration of multiple FQs in 20 (3.8%). Table 3 presents the ORs and 95% CIs for each outcome for patients in the combination therapy group who received LVFX or CPFX relative to those in the monotherapy group. There was no significant association with the likelihood of mortality or other complications between the combination therapy patients who used LVFX and the monotherapy patients. However, the combination therapy patients who used CPFX had a greater likelihood of mortality than did the monotherapy patients (adjusted OR = 25.98, 95% CI = 1.71–393.75).

**Table 3.** Subgroup analysis of the effect of LVFX and CPFX as part of combination therapy on each outcome with respect to monotherapy (*n* = 272) in patients with JSF

	LVFX (*N* = 457)			CPFX (*N* = 30)		
	*n* (%)	Unadjusted OR^a^	Adjusted OR^b^	*n* (%)	Unadjusted OR^a^	Adjusted OR^b^
Mortality						
Monotherapy	1 (0.4%)	1 (reference)	1 (reference)	1 (0.4%)	1 (reference)	1 (reference)
Combination therapy	7 (1.5%)	4.75 (0.56–40.07)	2.68 (0.28–25.56)	3 (10.0%)	30.11 (3.03–299.58)	25.98 (1.71–393.75)
Convulsions						
Monotherapy	4 (1.5%)	1 (reference)	1 (reference)	4 (1.5%)	1 (reference)	1 (reference)
Combination therapy	13 (2.8%)	1.96 (0.63–6.08)	1.84 (0.58–5.81)	2 (6.7%)	4.79 (0.84–27.31)	4.16 (0.64–27.20)
Upper gastrointestinal ulcers						
Monotherapy	22 (8.1%)	1 (reference)	1 (reference)	22 (8.1%)	1 (reference)	1 (reference)
Combination therapy	52 (11.4%)	1.46 (0.86–2.46)	1.11 (0.64–1.92)	4 (13.3%)	1.75 (0.56–5.46)	1.41 (0.43–4.56)
Diarrhoea						
Monotherapy	5 (1.8%)	1 (reference)	1 (reference)	5 (1.8%)	1 (reference)	1 (reference)
Combination therapy	3 (0.7%)	0.35 (0.08–1.49)	0.26 (0.06–1.18)	2 (6.7%)	3.81 (0.71–20.58)	2.75 (0.43–17.68)
Arrhythmia						
Monotherapy	5 (1.8%)	1 (reference)	1 (reference)	5 (1.8%)	1 (reference)	1 (reference)
Combination therapy	16 (3.5%)	1.94 (0.70–5.35)	1.64 (0.58–4.65)	1 (3.3%)	1.84 (0.21–16.31)	1.52 (0.16–14.34)
Hypoglycaemia						
Monotherapy	2 (0.7%)	1 (reference)	1 (reference)	2 (0.7%)	1 (reference)	1 (reference)
Combination therapy	1 (0.2%)	0.30 (0.03–3.28)	0.28 (0.03–3.12)	1 (3.3%)	4.66 (0.41–52.92)	3.19 (0.25–39.94)

A logistic regression model was used to calculate the adjusted OR with the 95% CI for mortality or complications by controlling for the above potential confounding factors. LVFX, levofloxacin; CPFX, ciprofloxacin; OR, odds ratio; Monotherapy, patients who received only a TC within one month of their JSF diagnosis; Combination therapy: patients who received both an FQ and a TC within one month of their JSF diagnosis and who used both drugs concurrently for more than one day.

^a^Unadjusted OR.

^b^ORs adjusted for sex, age group and severity.

We next investigated the concomitant use of NSAIDs for the treatment of JSF and its effect on the rate of convulsions. Fifty-eight of the 262 patients (22.1%) in the monotherapy group and 139 of the 502 patients (27.7%) in the combination therapy group received NSAIDs concomitantly with a TC or an FQ. Table 4 shows the ORs and 95% CIs for the likelihood of convulsions in the monotherapy patients who received NSAIDs (*n* = 58), the combination therapy patients who did not receive NSAIDs (*n* = 363), and the combination therapy patients who received NSAIDs (*n* = 139) relative to the monotherapy patients who did not receive NSAIDs (*n* = 204). A total of eight combination therapy patients who received NSAIDs experienced convulsions, among whom the median duration of the overlapping use of the FQ and NSAIDs was 4.5 days (IQR: 1.5–8.0). Compared with monotherapy patients who did not receive NSAIDs, combination therapy patients who received NSAIDs (adjusted OR: 5.44, 95% CI: 1.13–26.30), combination therapy patients who did not receive NSAIDs (adjusted OR: 2.55, 95% CI: 0.55–11.88) and monotherapy patients who received NSAIDs (adjusted OR: 1.74, 95% CI: 0.15–19.65) tended to develop convulsions more frequently in this order. Compared to monotherapy, combination therapy exhibited adjusted ORs of 3.12 (95% CI: 0.38–25.74) with NSAIDs and 2.55 (95% CI 0.55–11.88) without NSAIDs for the development of convulsions; however, the interaction was not significant (*P* = 0.88). In summary, combination therapy and NSAIDs were actually coadministered in the treatment of JSF, which tended to have a greater likelihood of convulsions, although there was insufficient evidence that the effect differed by the coadministration of NSAIDs.

**Table 4.** ORs and 95% CIs for the likelihood of convulsions in the tetracycline monotherapy and FQ-based combination therapy groups with and without the simultaneous use of NSAIDs

	*n* (%)	Unadjusted OR^a^	Adjusted OR^b^
Convulsions			
Monotherapy −NSAIDs (*n* = 204)	2 (1.0%)	1 (reference)	1 (reference)
Monotherapy +NSAIDs (*n* = 58)	1 (1.7%)	1.77 (0.16–19.89)	1.74 (0.15–19.65)
Combination therapy −NSAIDs (*n* = 363)	11 (3.0%)	3.16 (0.69–14.38)	2.55 (0.55–11.88)
Combination therapy+NSAIDs (*n* = 139)	8 (5.8%)	6.17 (1.29–29.50)	5.44 (1.13–26.30)

A logistic regression model was used to calculate the adjusted OR with the 95% CI for convulsions associated with combination therapy and NSAIDs by controlling for the above potential confounding factors. OR, odds ratio; NSAIDs, nonsteroidal anti-inflammatory drugs; −NSAIDs, without NSAIDs; +NSAIDs, with NSAIDs; monotherapy, patients receiving only a TC within one month of their JSF diagnosis; combination therapy, patients who received both an FQ and a TC within one month of their JSF diagnosis and who used both drugs concurrently for more than one day.

^a^Unadjusted OR.

^b^ORs adjusted for sex, age group and severity.

To minimize the impact of time-dependent covariates, we limited the combination therapy group to patients in which FQs and TCs were initiated simultaneously and conducted the same analyses; the conclusions remained unchanged. A total of 420 patients who simultaneously received FQs and TCs were categorized into the combination therapy group, and the patients in the combination therapy group were more likely to be 65 years or older [310 (73.8%) versus 160 (58.8%); *P* < 0.001], to be hospitalized [388 (92.4%) versus 192 (70.6%); *P* < 0.001], and to have severe disease [71 (16.9%) versus 17 (6.2%); *P* < 0.001] than were those in the monotherapy group (Supplementary Table 1, available as Supplementary data at *JAC* Online). There were no significant differences in clinical outcomes, including mortality, convulsions, upper gastrointestinal ulcers, diarrhoea, arrhythmia and hypoglycaemia, between the combination therapy and monotherapy groups (Supplementary Table 2). The likelihood of mortality was greater in patients receiving combination therapy with CPFX (*n* = 23) than in those receiving monotherapy (adjusted OR = 26.74, 95% CI = 1.83–389.93) (Supplementary Table 3). A total of 110 of the 408 patients (27.0%) in the combination therapy group received NSAIDs concomitantly with FQs; convulsions were more common among patients on combination therapy with NSAIDs than among those on monotherapy without NSAIDs (adjusted OR: 5.96, 95% CI: 1.20–29.55) (Supplementary Table 4).

## Discussion

In this retrospective cohort study, we assessed the disadvantages of using FQs combined with TCs for the treatment of JSF, which has not been previously evaluated, by employing a large database that included clinical information from DPC hospitals in Japan. This study revealed no significant improvement in the effectiveness of combination therapy for treating JSF. Furthermore, subgroup analyses demonstrated a positive association between CPFX-based combination therapy and mortality compared with monotherapy (adjusted OR = 25.98, 95% CI = 1.71–393.75) and the potential of combination therapy with NSAIDs to increase the likelihood of convulsions compared with monotherapy without NSAIDs (adjusted OR: 5.44, 95% CI = 1.13–26.30).

In terms of the benefits of combination therapy, this study did not demonstrate the superiority of combination therapy in terms of mortality for patients with JSF, as shown in Table 2, which is consistent with results previously reported in Japan.^6^ While a previous study reported an antipyretic effect of combination therapy,^5^ the effectiveness of this therapy should be assessed based on mortality because symptom duration is prone to bias and does not necessarily correlate with mortality.^14^ Combination therapy has not been shown to improve mortality in any previous study, and the necessity of adopting combination therapy has not been demonstrated in the context of therapeutic effectiveness.

In terms of the disadvantages of combination therapy, subgroup analysis revealed significantly greater mortality in the CPFX-based combination therapy group than in the monotherapy group after adjusting for sex, age group and severity as shown in Table 3. Three deaths occurred among thirty subjects; this number was insufficient to establish a definitive association, but these results suggest that compared with monotherapy, CPFX-based combination therapy may have a negative influence on mortality. Although the exact reason for the observed mortality outcomes could not be definitively determined, there is a possibility that adverse effects associated with CPFX use might have influenced the results.^15^

A total of 139 of 502 patients (27.7%) in the combination therapy group received NSAIDs concomitantly with FQs, and combination therapy with NSAIDs was significantly more strongly associated with a risk of convulsions than monotherapy without NSAIDs, as shown in Table 4. Although an interaction between combination therapy and NSAID use was not evident in the present study, the combination of FQs and NSAIDs is known to be a risk factor for seizures,^8,16,17^ and the trends shown in Table 4 are consistent with these findings. The use of FQs in addition to TCs leads to the unintentional combination of FQs and NSAIDs, which can cause convulsions as a side effect. NSAIDs are known for their side effects, including renal, gastrointestinal and cardiovascular complications,^18–20^ and therefore, alternatives such as paracetamol are more appropriate for antipyretic and analgesic use during the clinical course of JSF. Both the combination therapy and the frequent use of NSAIDs may indicate an underlying issue with the appropriate use of medications.

Considering that it did not improve mortality but instead revealed unfavourable effects, the combination of TCs and FQs should be discouraged. Clinicians often prioritize the advantages of combining drugs; however, it is crucial to give equal consideration to the disadvantages and harms of drug combinations.

Due to the rarity of JSF, recruiting a sufficient number of patients for a prospective comparative study is challenging. Therefore, we analysed a substantial number of diagnosed patients using the MDV database. This study is the first to specifically investigate the disadvantages associated with combination therapy for JSF treatment.

This study has several limitations. First, there are some concerns regarding the nature of the MDV database used in this study. Confounders and covariates may not have been fully controlled for and therefore may not have provided a comprehensive explanation of the outcomes in this retrospective cohort study. For example, while we defined severe JSF cases and made statistical adjustments for severity, there might have been other potential confounders and covariates, such as comorbidities or the virulence of the JSF strain, affecting these severe cases. Although *Rickettsia japonica* strains collected from multiple sources across Japan exhibit remarkably low genomic diversity,^21^ further confirmation with a more balanced distribution of severity is desirable. Target cases were identified in the MDV database by a disease code search, which may raise suspicions of inadequate diagnostic accuracy. The accuracy of the diagnoses could be reasonably assured because JSF patients are carefully monitored in accordance with the provisions in Japan, and only patients with confirmed cases of the disease were examined in this study. Nevertheless, the extent to which confirmed cases adhered to the diagnostic criteria and the reasons why suspected cases did not meet these criteria were uncertain. Because the data related to diagnoses also included a monthly data, we defined treatment drugs as those prescribed ‘within one month of receiving their JSF diagnosis’. This approach made it challenging to specify the timing of prescriptions more precisely. The MDV database has the potential for selection bias, suggesting that the study subjects might not accurately represent the actual patient population. There is a possibility that medical information from hospitals not covered by the MDV database remains unobserved. However, complications, which typically occur during treatment and are not usually long-term, can reasonably be assumed to have been documented within the same medical institution responsible for the patient’s treatment and therefore included in the MDV database. Second, there is another concern related to the definition of combination therapy. It has been reported that JSF often requires time for defervescence,^1^ and adding additional antibiotics during treatment could be a practical option if the fever persists. To mirror actual clinical practice, we classified patients for whom another antibiotic was added mid-course as part of the combination therapy group. However, not including the simultaneous initiation of TC and FQ in the definition of combination therapy may lead to bias due to changes in time-dependent covariates. Patients with unfavourable clinical courses are more likely to receive additional therapy mid-course, which could then be classified as combination therapy. Therefore, we have also added the condition of ‘simultaneous initiation of FQ and TC’ to the definition of combination therapy and confirmed that the conclusions remain unchanged, as shown in Supplementary Tables 1–4. Further integration and analysis of additional information may reveal an association between combination therapy and mortality that was not apparent in the present study. Additionally, supporting the conclusions drawn in this study through prospective research conducted in clinical settings is desirable.

Because comparative studies often lack a sufficient number of rare disease patients, there tends to be a reliance on expert opinions with insufficient evidence in clinical practice. In this study, we used a large database to examine not only the benefits but also the disadvantages of combination therapy, demonstrating that combination therapy may have potential disadvantages. A fair assessment that weighs both the benefits and disadvantages is essential for the appropriate use of antibiotics.

### Conclusions

This study provided no evidence that combination therapy with FQs and TCs improves outcomes in terms of mortality. Additionally, the concomitant use of CPFX and a TC was associated with an increased likelihood of mortality compared with the use of a TC alone. Moreover, combination therapy allows for the possibility of inappropriate coadministration of FQs and NSAIDs, which is known to be a risk factor for seizures. The results of this study indicated that combination therapy has deleterious effects.

## Supplementary Material

dkae192_Supplementary_Data

## Data Availability

The datasets analysed for this study are available from the corresponding author upon reasonable request.

## Supplementary data

Supplementary Tables 1 to 4 are available as Supplementary data at *JAC* Online.
