# Supplementary material for: Deleterious effects of a combination therapy using fluoroquinolones and tetracyclines for the treatment of Japanese spotted fever: a retrospective cohort study based on a Japanese hospital database
Source: J Antimicrob Chemother. 2024 Jun 12;79(8):1962–8. doi: 10.1093/jac/dkae192 (PMC11290883; doi:10.1093/jac/dkae192)
Supplement: dkae192_Supplementary_Data [file dkae192_supplementary_data.docx]

**Tables**

**Supplementary Table 1.** Basic characteristics of the study participants at the time of Japanese spotted fever diagnosis, with a focus on cases where combination therapy was initiated simultaneously.

|  |  | Total  (N = 692) | Monotherapy  (N = 272) | Combination therapy  (N = 420) | p value |
| --- | --- | --- | --- | --- | --- |
| Sex, (female), n (%) | | 354 (51.2%) | 134 (49.3%) | 220 (52.4%) | 0.44 |
| Age |  |  |  |  |  |
|  | Median age, years (IQR) | 71 (60–79) | 67 (53–77) | 73 (64–80) | <0.001 |
|  | Age group, (≥65), n (%) | 470 (67.9%) | 160 (58.8%) | 310 (73.8%) | <0.001 |
| Inpatient, n (%) | | 580 (83.8%) | 192 (70.6%) | 388 (92.4%) | <0.001 |
| Severe cases, n (%) | | 88 (12.7%) | 17 (6.2%) | 71 (16.9%) | <0.001 |

IQR: interquartile range; Monotherapy: patients who received only a TC within one month of their JSF diagnosis; Combination therapy: patients who initiated both an FQ and a TC simultaneously within one month of their JSF diagnosis and who used both drugs concurrently for more than one day. Patient characteristics were compared between the therapy groups using Fisher’s exact test for categorical variables or the Mann‒Whitney U test for continuous variables.

**Supplementary Table 2.** Odds ratios and 95% confidence intervals of tetracycline and fluoroquinolone combination therapy for each clinical outcome with respect to tetracycline monotherapy, with a focus on cases where combination therapy was initiated simultaneously.

|  |  | n (%) | Unadjusted OR^a^ | Adjusted OR^b^ |
| --- | --- | --- | --- | --- |
| Mortality | |  |  |  |
|  | Monotherapy (N = 272) | 1 (0.4%) | 1 (reference) | 1 (reference) |
|  | Combination therapy (N = 420) | 6 (1.5%) | 4.05 (0.48–33.88) | 2.28 (0.26–20.11) |
| Convulsions | |  |  |  |
|  | Monotherapy | 4 (1.5%) | 1 (reference) | 1 (reference) |
|  | Combination therapy | 16 (3.8%) | 2.65 (0.88–8.02) | 2.23 (0.72–6.88) |
| Upper gastrointestinal ulcers | |  |  |  |
|  | Monotherapy | 22 (8.1%) | 1 (reference) | 1 (reference) |
|  | Combination therapy | 48 (11.4%) | 1.47 (0.86–2.49) | 1.13 (0.65–1.96) |
| Diarrhoea | |  |  |  |
|  | Monotherapy | 5 (1.8%) | 1 (reference) | 1 (reference) |
|  | Combination therapy | 3 (0.7%) | 0.38 (0.09–1.62) | 0.28 (0.06–1.29) |
| Arrhythmia | |  |  |  |
|  | Monotherapy | 5 (1.8%) | 1 (reference) | 1 (reference) |
|  | Combination therapy | 14 (3.3%) | 1.84 (0.66–5.17) | 1.47 (0.51–4.26) |
| Hypoglycaemia | |  |  |  |
|  | Monotherapy | 2 (0.7%) | 1 (reference) | 1 (reference) |
|  | Combination therapy | 2 (0.5%) | 0.65 (0.09–4.61) | 0.47 (0.06–3.51) |

^a^Unadjusted odds ratio, ^b^Odds ratio adjusted for sex, age group and severity. A logistic regression model was used to calculate the adjusted odds ratio with the 95% confidence interval for mortality or complications by controlling for the above potential confounding factors. OR: odds ratio; Monotherapy: patients who received only TC within a month of their JSF diagnosis; Combination therapy: patients who initiated both an FQ and a TC simultaneously within one month of their JSF diagnosis and who used both drugs concurrently for more than one day.

**Supplementary Table 3.** Subgroup analysis of the effect of LVFX and CPFX as part of combination therapy on each outcome with respect to monotherapy (n = 272) in patients with Japanese spotted fever, with a focus on cases where combination therapy was initiated simultaneously**.**

|  |  | LVFX (N = 374) | | |  | CPFX (N = 23) | | |
| --- | --- | --- | --- | --- | --- | --- | --- | --- |
|  |  | n (%) | Unadjusted OR^a^ | Adjusted OR^b^ |  | n (%) | Unadjusted OR^a^ | Adjusted OR^b^ |
| Mortality | |  |  |  |  |  |  |  |
|  | Monotherapy | 1 (0.4%) | 1 (reference) | 1 (reference) |  | 1 (0.4%) | 1 (reference) | 1 (reference) |
|  | Combination therapy | 3 (0.8%) | 2.46 (0.26–23.74) | 1.77 (0.17–18.19) |  | 3 (13.0%) | 40.65 (4.04–408.82) | 26.74 (1.83–389.93) |
| Convulsions | |  |  |  |  |  |  |  |
|  | Monotherapy | 4 (1.5%) | 1 (reference) | 1 (reference) |  | 4 (1.5%) | 1 (reference) | 1 (reference) |
|  | Combination therapy | 12 (3.2%) | 2.22 (0.71–6.96) | 2.07 (0.65–6.61) |  | 2 (8.7%) | 6.38 (1.10–36.89) | 6.94 (0.95–50.65) |
| Upper gastrointestinal ulcers | |  |  |  |  |  |  |  |
|  | Monotherapy | 22 (8.1%) | 1 (reference) | 1 (reference) |  | 22 (8.1%) | 1 (reference) | 1 (reference) |
|  | Combination therapy | 39 (10.4%) | 1.32 (0.77–2.29) | 1.02 (0.58–1.81) |  | 3 (13.0%) | 1.70 (0.47–6.19) | 1.35 (0.35–5.17) |
| Diarrhoea | |  |  |  |  |  |  |  |
|  | Monotherapy | 5 (1.8%) | 1 (reference) | 1 (reference) |  | 5 (1.8%) | 1 (reference) | 1 (reference) |
|  | Combination therapy | 0 (0.0%) | NA | NA |  | 2 (8.7%) | 5.09 (0.93–27.81) | 3.65 (0.52–25.50) |
| Arrhythmia | |  |  |  |  |  |  |  |
|  | Monotherapy | 5 (1.8%) | 1 (reference) | 1 (reference) |  | 5 (1.8%) | 1 (reference) | 1 (reference) |
|  | Combination therapy | 12 (3.2%) | 1.77 (0.62–5.08) | 1.53 (0.52–4.51) |  | 1 (4.3%) | 2.43 (0.27–21.70) | 1.87 (0.19–18.10) |
| Hypoglycaemia | |  |  |  |  |  |  |  |
|  | Monotherapy | 2 (0.7%) | 1 (reference) | 1 (reference) |  | 2 (0.7%) | 1 (reference) | 1 (reference) |
|  | Combination therapy | 1 (0.3%) | 0.36 (0.03–4.01) | 0.33 (0.03–3.69) |  | 1 (4.3%) | 6.14 (0.54–70.37) | 3.76 (0.29–48.47) |

^a^Unadjusted odds ratio, ^b^Odds ratio adjusted for sex, age group and severity. A logistic regression model was used to calculate the adjusted odds ratio with the 95% confidence interval for mortality or complications by controlling for the above potential confounding factors. LVFX: levofloxacin; CPFX: ciprofloxacin; OR: odds ratio; Monotherapy: patients who received only a TC within one month of their JSF diagnosis; Combination therapy: patients who initiated both an FQ and a TC simultaneously within one month of their JSF diagnosis and who used both drugs concurrently for more than one day.

**Supplementary Table 4.** Odds ratios and 95% confidence intervals for the likelihood of convulsions in the tetracycline monotherapy and fluoroquinolone-based combination therapy groups with and without the simultaneous use of nonsteroidal anti-inflammatory drugs, with a focus on cases where combination therapy was initiated simultaneously**.**

|  |  | n (%) | Unadjusted OR^a^ | Adjusted OR^b^ |
| --- | --- | --- | --- | --- |
| Convulsions | |  |  |  |
|  | Monotherapy -NSAIDs  (n = 204) | 2 (1.0%) | 1 (reference) | 1 (reference) |
|  | Monotherapy +NSAIDs  (n = 58) | 1 (1.7%) | 1.77 (0.16–19.89) | 1.74 (0.15–19.61) |
|  | Combination therapy  -NSAIDs  (n = 298) | 9 (3.0%) | 3.15 (0.67–14.71) | 2.52 (0.53–12.04) |
|  | Combination therapy  +NSAIDs  (n = 110) | 7 (6.4%) | 6.86 (1.40–33.64) | 5.96 (1.20–29.55) |

^a^Unadjusted odds ratio, ^b^Odds ratio adjusted for sex, age group and severity. A logistic regression model was used to calculate the adjusted odds ratio with the 95% confidence interval for convulsions associated with combination therapy and NSAIDs by controlling for the above potential confounding factors. OR: odds ratio; NSAIDs: nonsteroidal anti-inflammatory drugs; -NSAIDs: without NSAIDs; +NSAIDs: with NSAIDs; monotherapy: patients receiving only a TC within one month of their JSF diagnosis; combination therapy: patients who initiated both an FQ and a TC simultaneously within one month of their JSF diagnosis and who used both drugs concurrently for more than one day.
